# Supplementary figures and images for: Expected length of stay in residential aged care facilities in Australia: Assessing the impact of dementia using machine learning
Source: PLoS One. 2025 May 16;20(5):e0321612. doi: 10.1371/journal.pone.0321612 (PMC12083792; doi:10.1371/journal.pone.0321612)

**Supplementary Table 1: ACFI Domains and their Constituent Characteristics**

**
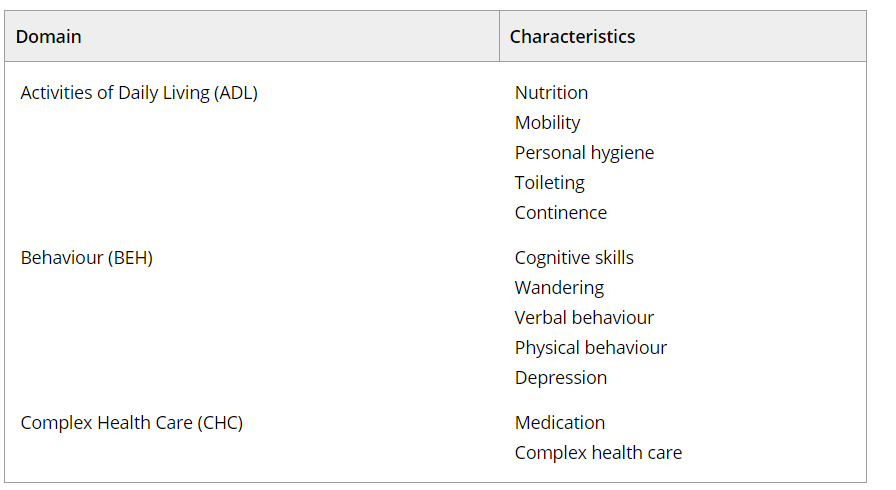
**

Supplement: S1 Table — (DOCX) [file pone.0321612.s001.docx]
