# Supplementary material for: Expected length of stay in residential aged care facilities in Australia: Assessing the impact of dementia using machine learning
Source: PLoS One. 2025 May 16;20(5):e0321612. doi: 10.1371/journal.pone.0321612 (PMC12083792; doi:10.1371/journal.pone.0321612)
Supplement: S2 Table — (DOCX) [file pone.0321612.s002.docx]

**Supplementary Table 2: Model Comparison Summary**

| Model | RMSE | *R^2^* |
| --- | --- | --- |
| Random Forest | 594.46 | 24.90% |
| Gradient Boosting | 573.64 | 30.22% |
| Linear Regression | 625.55 | 16.8% |

Abbreviation: RMSE Root mean square error
