# Supplementary material for: Expected length of stay in residential aged care facilities in Australia: Assessing the impact of dementia using machine learning
Source: PLoS One. 2025 May 16;20(5):e0321612. doi: 10.1371/journal.pone.0321612 (PMC12083792; doi:10.1371/journal.pone.0321612)
Supplement: Attachment — Submitted filename: PLOS ResponsetoReviewer_9 Jan 25.docx [file pone.0321612.s004.docx]

The paper discusses modelling of Length of Stay (LOS) of elderly population in Australia.

1. The rule of thumb is that Machine Learning (ML) is applicable after the authors demonstrate that simple and well-interpretable classic methods do not work. Although the authors report R-squared I have to remind that this metric increases with the number of parameters and model complexity in general. This means that R-squared is not a fair metric to compare linear regression and the Random Forest regression. Since the authors aim to predict LOS another metric, such as the Root Mean Square of LOS prediction via the leave-one-out technique should be employed.

Response: Thank you very much for your thoughtful feedback. We did indeed use cross-validation to determine the best-performing model. The *R*^2^ value was included solely for in-sample comparison, as it may be of interest to readers wishing to understand the proportion of variation explained by the model, specifically for the linear regression. However, the final model selection was based on the Root Mean Square Error (RMSE) derived from a 3-fold cross-validation process to ensure a rigorous assessment of model performance. To further clarify this point, we have updated the first paragraph of Section 3.2 accordingly.

2. The difference between states is not addressed. Mixed model with random intercepts that represent the state-specific LOS is easy to apply, and I recommend employing this model. I wonder how the state-specific LOS can be accounted using the ML methodology.

Response: Thank you for your insightful feedback and suggestions.

As illustrated in Figure 3, Table 1, and also elaborated in the Discussion section of the paper, there were no distinctive differences among states in terms of age and sex distributions or the average length of stay (LOS). This findings is also shown in Table 2 where the effect of state/territories was not statistically significant at the 1% level.

Regarding the use of machine learning models, the state/territories variable has been incorporated as a categorical feature. The results aligned closely with our expectations and the linear regression outcomes, showing contribution of state/territories importance remains minimal and close to zero.

The nonlinearity of LOS versus Age is not addressed in regression analysis. I suggest showing the cross plot (Age,LOS) and displaying linear, quadratic, and a nonparametric regression (such as ‘loess’ in R) to understand how nonlinear the relationship is. This univariate analysis must be repeated for all other predictors.

Response: Thank you for your thoughtful feedback and constructive suggestions.

In the revised manuscript, we have now included figures depicting the average length of stay (LOS) versus year, age, Activities of daily living **(**ADL), and Behavioral (BEH) need scores in the revised paper (please refer to Figures 6-8). The curves presented in these figures utilize the LOWESS smoothing method, as you recommended, and suggest potential non-linearity in these relationships.

To address this observation more rigorously, we have updated the linear regression model by incorporating natural splines for year, age, ADL, and BEH scores. This allows for greater flexibility in capturing non-linear patterns while maintaining interpretability. Please note that the remaining variables in the model are categorical and have been modelled using indicator variables. The differences in average LOS across the levels of these categorical variables are already presented in Table 1, making additional graphical representation redundant for this purpose.

Furthermore, Table 2 has been updated to reflect the results from the revised model. For the four continuous variables mentioned, significance tests have been provided for the overall effect of the spline terms. However, the coefficients for individual spline components are not reported, as they lack straightforward interpretability.

3. The authors must explain/interpret ‘Importance’ of the variables in Figs 4 and 5. Importance with respect to what? How to explain Importance to Medical Doctors? Why Importance scale is different in the figures?

Response: We have clarified in the revised paper that the term "importance" presented in Figures 4 and 5 were specifically referring to permutation importance, a widely used model-agnostic technique in machine learning for assessing the contribution of individual variables in predictive models.

It measures the effect of shuffling a feature on the model’s performance specifically the process involves training the model on the original data, computing the performance metric (e.g., RMSE), and then permuting each feature one at a time. After permuting a feature, the performance metric is recomputed, and the importance of the feature is calculated as the difference between the original and permuted performance scores. The formula for the importance of a feature is:

Importance of feature=Performance (original)−Performance (permuted)

The larger the drop in performance after permuting a feature, the more important that feature is for the model's predictions.

To make it clearer, we have added additional clarification regarding the concept of permutation importance in Section 3.1 in the revised manuscript to ensure greater clarity for readers.

4. Data are not available: “No - some restrictions will apply.” It’s unclear why de-identified data cannot be accessed. I have to remind the authors that the data availability is a major condition for research transparency and results reproducibility.

Response: The dataset used in this study consists of individual records organized and maintained by the Australian Institute of Health and Welfare (AIHW). Access to this dataset is restricted and granted only to approved individuals to ensure privacy and data security. Interested users may contact the AIHW directly to inquire about obtaining access to the de-identified data under the appropriate permissions.
