# Supplementary material for: Expected length of stay in residential aged care facilities in Australia: Assessing the impact of dementia using machine learning
Source: PLoS One. 2025 May 16;20(5):e0321612. doi: 10.1371/journal.pone.0321612 (PMC12083792; doi:10.1371/journal.pone.0321612)
Supplement: Attachment — Submitted filename: PLOS _ D 24_53339R1 ResponseReviewer_25Feb25.docx [file pone.0321612.s005.docx]

Overall response:

We thank the reviewer for the comments, and we apologise for the misleading conclusions that were actually drawn from our figures in the previous submission. The purpose of presenting Figures 6-8 in the earlier manuscript was to illustrate the relationship between LOS (Y variable) and explanatory variables (i.e., Year, BEH and ADL scores).

Due to our large sample size (n=509,142), we realise that it is not efficient or possible to show the complete scatter plot with the full dataset. As such, in the previously figures, we plotted an average LOS for each unique value of an explanatory variable. The LOWESS curve was applied to the resulting average LOS shown in each figure, and these curves are not necessarily consistent with that could be obtained using the complete dataset.

We have now clarified and reproduced our four plots in the current revised manuscript. In each plot, the LOWESS curve is obtained using the complete dataset, and the scatter points consist of 5,000 randomly selected subsample from the complete dataset. These scatter points should only be used as a snapshot of the full sample, and the non-linear patterns shown by the LOWESS curves are more important. In these new figures, it is clear that each of the four continuous variables may non-linearly influence the LOS. The paper has been revised to highlight these updates.

In addition, we would like to restate our logic and steps of our analyses:

1. Provide plots of LOS vs explanatory variables to preliminarily analyse the potential relationships.

2. The observed non-linear relationships preliminarily support the potential usefulness of ML models, which can accommodate such non-linearity without having to specify it. In comparison, the four continuous variables are modelled via splines in the usual linear regression to accommodate non-linearity.

3. Both linear regression and ML models support the significance of ADL and BEH scores.

4. ML models outperforms the linear regression as evidenced by RMSE and (pseudo) R-square, and thus is recommended as the final models to study and project LOS in our present study.

We believe that these new results can effectively address the previous concerns. Therefore, the three comments may no longer be applicable to our revised paper. Nevertheless, we decided to provide point-to-point response for the completeness.

1. Why in Fig. 6 Y=Average LOS has discrete values and in Figs 7-8 close to continuous?

Response: Thank you for your comments. In these figures, we plotted the average LOS for each unique value of the corresponding explanatory variables. Discrete values were found for Year since there were only 13 distinct values. In contrast, the other explanatory variables (ADL and BEH scores in Figures 7 and 8, respectively) had many more unique values and thus appeared to be continuous data.

2. Variables ADL Score and BEH Score have a weak relationship with the Y variable. It looks like the regression line is horizontal. The Y and the BEH score can be modeled as a stepwise function. All other variables presented in Fig. 6 are not significant.

Response: We thank the reviewer for highlighting this. As outlined above, these figures showed the LOWESS curve of unique values of each explanatory variable against the corresponding average LOS. They do not represent the true curve that may be obtained using the full sample. To more systematically justify the significance, the p-value in linear regression and importance scores in ML models should be used instead. In both cases, the ADL Score and BEH Score are believed to be significant and/or among the most important explanatory variables.

3. As far as the modeling of Y is concerned, the yellow line (a spline?) is the answer with R-squared hard to beat. I suspect that a quadratic function of Year on the log scale of Y can be as good as the yellow line.

Response: Thank you for your comment. While a quadratic model might seem to capture the non-linearity observed in the scatter plot, we chose to use splines in linear regression because it is more general and flexible. Splines allow the relationship to vary across different ranges of the explanatory variable, without imposing the fixed shape that a quadratic function does. This flexibility is especially useful if the true relationship deviates from a simple quadratic form, especially when other explanatory variables are later included in the full model.

Additionally, the ML models employed are designed to capture complex non-linear patterns without the need to specify a particular functional form in advance. Their ability to learn directly from the data means that they can adapt to various underlying relationships, including but not limited to linear, quadratic and other patterns.

In other words, our approaches (spline in linear regression and ML models) minimize the risk of model misspecification and allow for a better fit if the true relationship is more complex than a simple quadratic. More importantly, as evidenced by (pseudo) R-square and RMSE, ML models outperform the linear regression. This validates the reason that they are chosen as the final model in this paper.
